# Intraoperative measurement of the respiratory exchange ratio predicts postoperative complications after liver transplantation

**DOI:** 10.1186/s12871-022-01949-2

**Published:** 2022-12-28

**Authors:** Sean Coeckelenbergh, Olivier Desebbe, François Martin Carrier, Francois Thepault, Cécile De Oliveira, Florian Pellerin, Cyril Le Canne, Laurence Herboulier, Edita Laukaityte, Maya Moussa, Leila Toubal, Hiromi Kato, Hung Pham, Stephanie Roullet, Marc Lanteri Minet, Youssef Amara, Salima Naili, Oriana Ciacio, Daniel Cherqui, Jacques Duranteau, Jean-Louis Vincent, Philippe Van der Linden, Alexandre Joosten

**Affiliations:** 1 Department of Anesthesiology and Intensive Care, Paul Brousse Hospital, Assistance Publique - Hôpitaux de Paris, Université Paris-Saclay, Villejuif, France; 2 Outcomes Research Consortium, Cleveland, OH USA; 3 Department of Anesthesiology and Perioperative Medicine Sauvegarde Clinic, Ramsay Santé, Lyon, France; 4 Department of Anesthesiology and Department of Medicine, Critical Care Division, Centre hospitalier de l’Université de Montréal, Montréal, Québec, Canada; 5 Department of Liver Hepatobiliary Surgery & Liver Transplantation, Paul Brousse Hospital, Assistance Publique - Hôpitaux de Paris, Université Paris-Saclay, Villejuif, France; 6 Department of Intensive Care, Erasme University Hospital, Université Libre de Bruxelles, Brussels, Belgium; 7 Department of Anesthesiology, Grand Hôpital de Charleroi, Charleroi, Belgium

**Keywords:** Morbidity, Hemodynamic monitoring, Tissue hypoxia, Anaerobic metabolism, Shock

## Abstract

**Background:**

During surgery, any mismatch between oxygen delivery (DO_2_) and consumption (VO_2_) can promote the development of postoperative complications. The respiratory exchange ratio (RER), defined as the ratio of carbon dioxide (CO_2_) production (VCO_2_) to VO_2_, may be a useful noninvasive tool for detecting inadequate DO_2_. The primary objective of this study was to test the hypothesis that RER measured during liver transplantation may predict postoperative morbidity. Secondary objectives were to assess the ability of other variables used to assess the DO_2_/VO_2_ relationship, including arterial lactate, mixed venous oxygen saturation, and veno-arterial difference in the partial pressure of carbon dioxide (VAPCO_2_gap), to predict postoperative complications.

**Methods:**

This retrospective study included consecutive adult patients who underwent liver transplantation for end stage liver disease from June 27th, 2020, to September 5th, 2021. Patients with acute liver failure were excluded. All patients were routinely equipped with a pulmonary artery catheter. The primary analysis was a receiver operating characteristic (ROC) curve constructed to investigate the discriminative ability of the mean RER measured during surgery to predict postoperative complications. RER was calculated at five standardized time points during the surgery, at the same time as measurement of blood lactate levels and arterial and mixed venous blood gases, which were compared as a secondary analysis.

**Results:**

Of the 115 patients included, 57 developed at least one postoperative complication. The mean RER (median [25–75] percentiles) during surgery was significantly higher in patients with complications than in those without (1.04[0.96–1.12] vs 0.88[0.84–0.94]; *p* < 0.001). The area under the ROC curve was 0.87 (95%CI: 0.80–0.93; *p* < 0.001) with a RER value (Youden index) of 0.92 giving a sensitivity of 91% and a specificity of 74% for predicting the occurrence of postoperative complications. The RER outperformed all other measured variables assessing the DO_2_/VO_2_ relationship (arterial lactate, SvO_2_, and VAPCO_2_gap) in predicting postoperative complications.

**Conclusion:**

During liver transplantation, the RER can reliably predict postoperative complications. Implementing this measure intraoperatively may provide a warning for physicians of impending complications and justify more aggressive optimization of oxygen delivery. Further studies are required to determine whether correcting the RER is feasible and could reduce the incidence of complications.

**Supplementary Information:**

The online version contains supplementary material available at 10.1186/s12871-022-01949-2.

## Background

Patients receiving a liver transplant are at high-risk of developing intraoperative tissue hypoxia, which may lead to postoperative complications. Quantifying any potential mismatch between oxygen delivery (DO_2_) and oxygen consumption (VO_2_) in this population is therefore of particular interest [1, 2]. Various techniques have been used in an attempt to identify DO_2_/VO_2_ mismatch during general anesthesia [3], most notably by measuring arterial lactate concentrations, mixed venous oxygen saturation (SvO_2_) [4], and veno-arterial difference in the partial pressure of carbon dioxide (VAPCO_2_gap) [5]. In particular, blood lactate values are of great importance as they help guide therapeutic interventions during major abdominal surgery. Hyperlactatemia is frequent during major surgery [6], and is associated with postoperative complications, increased length of stay, and mortality [7]. Strategies to increase DO_2_ using goal-directed hemodynamic therapy are therefore highly recommended by anesthesia societies [8]. Blood lactate concentrations could be used as a marker, but have the limitation of being affected by various factors, including liver function, which is altered during liver transplantation surgery. In addition, changes in blood lactate concentrations are quite slow so that its measurement provides only an intermittent assessment of cellular function.

The respiratory exchange ratio (RER), on the other hand, may provide a more continuous indication of the presence of anaerobic metabolism in mechanically ventilated patients [1, 2]. The RER is calculated using values derived from the standard anesthesia machine gas analyzer with the following formula: RER = (FeCO_2_ - FiCO_2_) / (FiO_2_ - FeO_2_). These variables can be easily measured in any patient receiving mechanical ventilation. In open and laparoscopic abdominal surgery, the RER has been shown to detect hyperlactatemia and to predict postoperative complications with moderate accuracy [1, 2, 9]. However, little is known about the capacity of RER to predict postoperative complications during liver transplantation. The aim of this study was to determine whether an increased RER value during liver transplantation would predict postoperative complications. Secondary objectives were to compare this indicator with other variables assessing the DO_2_/VO_2_ relationship, including arterial blood lactate, SvO_2_, and VAPCO_2_gap.

## Methods

### Design and participants

All consecutive patients undergoing liver transplantation at Paul Brousse hospital in Villejuif, France from June 27th, 2020 to September 5th, 2021 were considered for inclusion in this cohort study. We excluded those who underwent urgent transplantation for acute liver failure, those who were not monitored with a pulmonary artery catheter, and patients with missing data on variables required to calculate the RER. We report our work using STROBE guidelines. The ethics committee of the French society of anesthesiology approved the study on June 8th, 2022 under the number IRB 00010254–2022-076 (Principal Investigator: Alexandre Joosten), and the IRB waived the need to obtain individual consent.

### Anesthesia protocol

All patients had at least one large peripheral venous catheter and a central multilumen access venous catheter. They were monitored according to the standards of the American Society of Anesthesiology (ASA) (i.e., pulse oximetry, non-invasive blood pressure, 5-lead EKG, inhaled and expired gases, and temperature monitoring), and had invasive blood pressure monitoring through a radial or a femoral arterial catheter. Pulmonary artery pressure, continuous cardiac index, and SvO_2_ were measured using a pulmonary artery catheter, inserted following anesthetic induction using the multilumen access catheter. Frontal electroencephalogram monitoring with the Bispectral index and other supplemental monitoring tools were used at the discretion of the attending anesthetist.

Following anesthesia induction with propofol or etomidate, sufentanil was used to control pain. Neuromuscular blockade was induced with succinylcholine (or rocuronium if contraindicated), and maintained with atracurium. Anesthesia was maintained using sevoflurane. Mean arterial pressure was maintained at least at 70 mmHg using a norepinephrine infusion.

The surgical technique was also standardized, including the so-called “3-vein piggy-back” technique for vena cava reconstruction. In rare cases of vena cava replacement, a veno-venous bypass was used in the presence of poor hemodynamic tolerance during caval clamping.

### Exposure

Our main exposure of interest was the RER, calculated using its determinants (FiO_2,_ FiCO_2_, FeO_2_, and FeCO_2_) at five standardized time points during surgery: T1, pulmonary artery catheter calibration; T2, vena cava clamping; T3, 10 minutes after portal reperfusion; T4, 10 minutes after arterial reperfusion; T5, end of surgery.

### Outcomes

The primary objective was to assess the RER’s capacity to predict a composite outcome of postoperative morbidity defined as the presence of at least one predefined postoperative complication occurring within 30 days after surgery and including sepsis, stroke, acute respiratory distress syndrome, myocardial infarction, wound dehiscence, biliary complications (both non-anastomotic and anastomotic strictures), acute kidney injury (KDIGO stage 2–3), vascular complications (hepatic artery stenosis or thrombosis, portal vein thrombosis, hepatic vein thrombosis), atrial fibrillation, primary graft non-function, reoperation for any cause, and death.

Secondary objectives were to assess the accuracy of blood lactate, SvO2, and VAPCO2gap to predict the composite outcome of postoperative morbidity. These variables were simultaneously measured with the RER via arterial and mixed venous blood gas sampling.

### Data collection

Patient baseline characteristics, intraoperative variables, postoperative complications, and 30-day mortality were prospectively collected by research staff using data from our electronic medical records.

### Statistical analysis

The Kolmogorov Smirnov test determined that data were not normally distributed and continuous variables are thus reported as median with quartiles [25th -75th percentile] and compared with a Mann-Whitney U-test. Discrete data are expressed as number and percentage and were compared using a Chi square or a Fisher’s exact test when indicated. A *p* value inferior to 0.05 was considered statistically significant, unless multiple comparisons were carried out, as was the case when comparing different time points. In that situation the p value was adjusted for multiple comparisons with the Bonferroni correction (*p* = 0.05/5 = 0.01) and was thus significant when < 0.01.

Our primary analysis was the estimation of the discriminative property of the mean RER calculated from the five time points to predict postoperative complications using the area under the receiver operating characteristics (AUROC) curve. To do this, we separated patients into two groups: those with and those without complications. We first fitted a logistic regression model and then estimated the AUROC according to Delong et al. and its 95% confidence intervals with the calculation of an exact Binomial Confidence Interval [10]. From the ROC curves, the optimal cut-off value yielding the greatest combined sensitivity and specificity was measured using the Youden index. We defined values within the 95% CI of the obtained threshold value as inconclusive (gray zone) according to Cannesson et al. [11]. This gray zone approach defines a zone of uncertainty, which explores the clinical usefulness of the RER to predict postoperative complications. Statistical analyses were conducted with MedCalc® Statistical Software version 19.6.4 (MedCalc Software Ltd., Ostend, Belgium; https://www.medcalc.org; 2021).

## Results

**Fig. 1:**
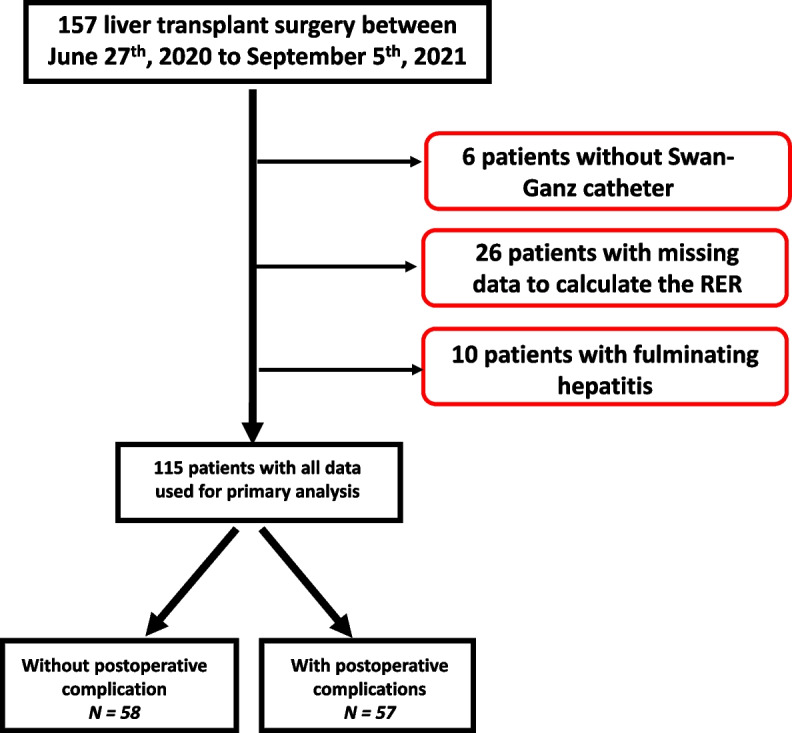
Flow chart

Among the 157 patients undergoing liver transplantation during the study period, 42 were excluded because they had urgent transplantation for fulminating hepatitis (*N* = 10), missing data to determine the RER (*N* = 26), or no pulmonary artery catheter (*N* = 6). Of the remaining 115 patients, 57 (50%) developed at least one postoperative complication (Fig. 1**),** predominantly infectious, renal, or reoperation (Table 1**)**. As patients with missing data were excluded, there was no missing data.

**Table 1:** Postoperative complications

Type of complication, N (%)	
Infection including sepsis	30 (26.1)
Stroke	6 (5.2)
ARDS	8 (7.0)
Myocardial infarction	1 (0.9)
Wound dehiscence	3 (2.6)
Reoperation	17 (14.8)
Biliary complications	4 (3.5)
Acute kidney injury^a^	26 (22.6)
Vascular complications	8 (7.0)
Atrial fibrillation	1 (0.9)
Primary graft non-function	11 (9.6)
Death	1 (0.9)

ARDS: acute respiratory distress syndrome. a Acute kidney injury includes KDIGO stages 2 and 3

**Fig. 2:**
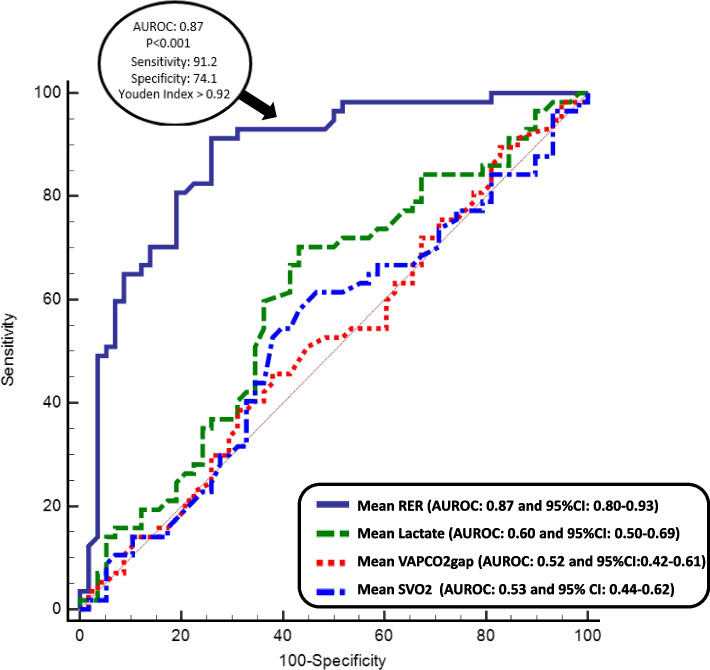
Receiver operating characteristic curve to examine the ability of the RER, VAPCO_2_gap, lactate and SvO_2_ can predict the occurrence of postoperative complications

A mean RER of 0.92 (95% CI: 0.91–1.02) predicted the occurrence of postoperative complications with a sensitivity of 91% and a specificity of 74%. The AUROC was 0.87 (95% CI: 0.80–0.93; *p* < 0.001). 24 patients (21% of the study group) were in the gray zone (0.92–0.99). The RER outperformed all other indicators of tissue perfusion (Fig. 2).

Patients who developed postoperative complications had similar baseline characteristics to those who did not **(**Table 2**)** but stayed longer in the intensive care unit (149 hours vs 104 hours; *p* = 0.004) and in the hospital (36 days vs 23 days; *p* = 0.002). 30-day mortality was not statistically significantly different between the two groups. Amounts of intraoperative fluids, blood loss, and operative times were also similar in the two groups (Supplementary Table 1).

**Table 2:** Preoperative data

	No Complication (***n*** = 58)	Complication (***n*** = 57)	***P***-value
**Baseline data; N (%)**			
• Age (years)	58 [51–64]	57 [47–65]	0.679
• Height (cm)	173 [162–178]	172 [162–176]	0.949
• Weight (kg)	72 [64–85]	74 [63–89]	0.989
• Arterial hypertension, N (%)	17 (29)	20 (17.4)	0.553
• Dyslipidemia, N (%)	4 (7)	4 (7)	> 0.999
• Type 1 diabetes, N (%)	2 (4)	0 (0)	0.496
• Type 2 diabetes, N (%)	3 (5)	1 (1)	0.619
• Atrial fibrillation, N (%)	14 (24)	16 (28)	0.675
• Peripheral arteriopathy, N (%)	6 (10)	5 (9)	> 0.999
• Heart failure, N (%)	5 (9)	9 (16)	0.268
• Chronic renal failure, N (%)	3 (5)	1 (2)	0.317
• Asthma, N (%)	0 (0)	3 (5)	0.119
• COPD, N (%)	2 (3)	1 (2)	0.569
**Transplant-specific history**			
• Model for end-stage liver disease score	18 [12–25]	14 [9–22]	0.133
• Retransplant, N (%)	2 (4)	3 (4)	> 0.999
• Combined liver-kidney transplant, N (%)	0 (0)	2 (4)	0.244
• Cold ischemia time (min)	446 [345–525]	431 [356–528]	0.922
• Warm ischemia time (min)	435 [362–555]	452 [377–567]	0.452
• Graft weight (kg)	1350 [1148–1545]	1310 [1000–1500]	0.475
**Indication for transplantation, n (%)**			0.159
• Hepatocellular carcinoma	5 (9)	4 (7)	
• Alcoholic cirrhosis	33 (57)	30 (53)	
• Non-alcoholic steato hepatitis cirrhosis	8 (14)	2 (4)	
• Viral cirrhosis	3 (5)	5 (9)	
• Other^a^	9 (16)	16 (28)	

Data are presented as medians with (first and third quartiles [q1, q3]) or frequencies with proportions in (%). a Includes primary sclerosing cholangitis, amyloidosis, primary biliary cholangitis, autoimmune hepatitis, and overlapping syndromes. COPD: Chronic Obstructive Pulmonary Disease

**Fig. 3:**
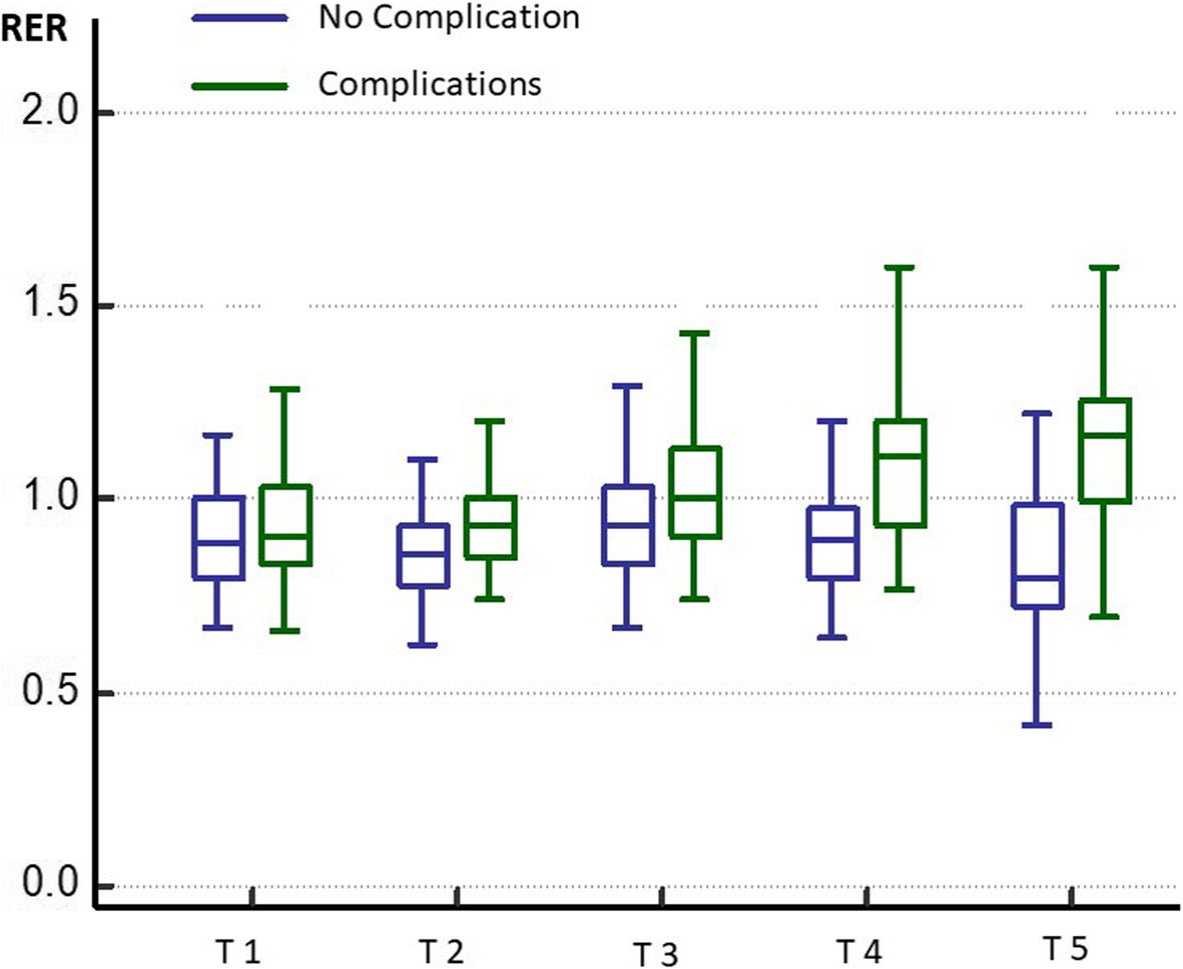
Evolution of the RER at the 5 different time points between patients who developed postoperative complications and those who did not. T1 = PAC Calibration - T2 = Caval Clamping - T3 = Post Portal Reperfusion - T4 = Post Arterial reperfusion - T5 = End of surgery

The evolution of RER values over time was significantly different in the two groups of patients (Fig. 3). The RER increased steadily after vena cava clamping until the end of surgery in patients who developed postoperative complications, but remained almost unchanged in those who did not. Changes in blood lactate levels over time were also significantly different between the two groups of patients but occurred later. There were no significant differences in changes in SvO_2_ or VAPCO_2_gap** (**Table 3**).**

**Table 3:** Intraoperative hemodynamic and tissue perfusion indices

	Without Complication (n = 58)	With Complications (n = 57)	***P*** value
**Respiratory Exchange Ratio**			
* T1*	0.89 [0.80–1.00]	0.90 [0.83–1.03]	0.242
* T2*	0.86 [0.78–0.93]	0.93 [0.85–1.00]	0.005
* T3*	0.93 [0.83–1.03]	1.00 [0.90–1.13]	0.015
* T4*	0.90 [0.80–0.98]	1.11 [0.93–1.20]	< 0.001
* T5*	0.80 [0.72–0.99]	1.17 [1.00–1.26]	< 0.001
** →Average of T1 to T5**	0.88 [0.84–0.94]	1.04 [0.96–1.12]	< 0.001
**Mean Arterial Pressure (mmHg)**			
* T1*	82 [73–86]	83 [74–88]	0.489
* T2*	76 [70–88]	73 [68–86]	0.354
* T3*	83 [77–88]	79 [74–89]	0.467
* T4*	78 [72–92]	78 [71–84]	0.317
* T5*	73 [68–82]	75 [69–80]	0.643
** →Average of T1 to T5**	79 [76–82]	78 [75–84]	0.741
**Cardiac Index (l/min/m2)**			
* T1*	4.2 [3.2–5.4]	4.3 [3.4–5.7]	0.667
* T2*	3.7 [3.1–4.8]	4.3 [3.1–5.7]	0.275
* T3*	4.0 [3.3–5.0]	4.1 [3.5–5.8]	0.159
* T4*	4.0 [2.8–5.5]	4.1 [3.3–4.8]	0.662
* T5*	5.1 [4.2–6.5]	5.5 [4.2–6.5]	0.519
** →Average of T1 to T5**	4.3 [3.6–5.2]	4.5 [3.7–5.7]	0.322
**SVRI (dynes. s. m**^**2**^**/cm**^**5**^**)**			
* T1*	1427 [1027–1802]	1248 [825–1781]	0.278
* T2*	1379 [1153–1720]	1244 [996–1633]	0.196
* T3*	1351 [1065–1844]	1223 [934–1873]	0.457
* T4*	1505 [1103–2019]	1395 [1041–1795]	0.318
* T5*	963 [747–1234]	941 [786–1237]	0.973
** →Average of T1 to T5**	1325 [1019–1724]	1210 [916–1664]	0.444
**pCO2 gradient (mmHg)**			
* T1*	3.3 [2.5–4.6]	3.7 [2.6–4.7]	0.663
* T2*	3.6 [2.1–4.5]	3.5 [2.4–4.3]	0.904
* T3*	3.4 [2.2–4.5]	3.0 [2.0–4.5]	0.570
* T4*	3.6 [2.5–5.0]	4.0 [2.4–5.0]	0.724
* T5*	3.3 [2.8–4.3]	3.2 [2.2–3.9]	0.423
** →Average of T1 to T5**	3.6 [2.8–4.2]	3.5 [2.8–4.1]	0.733
**Lactate value (mmol/l)**			
* T1*	1.5 [1.0–2.0]	1.2 [0.9–1.6]	0.017
* T2*	2.4 [1.5–3.4]	2.1 [1.6–3.3]	0.650
* T3*	3.5 [2.8–4.5]	3.9 [2.9–5.0]	0.171
* T4*	4.4 [3.4–5.4]	5.2 [4.0–6.6]	0.012
* T5*	3.8 [2.6–5.6]	5.4 [3.8–7.3]	0.005
** →Average of T1 to T5**	3.1 [2.7–4.0]	3.6 [3.0–4.4]	0.058
**SvO**_**2**_ **(%)**			
* T1*	84 [79–88]	86 [80–88]	0.492
* T2*	82 [78–87]	82 [78–86]	0.924
* T3*	86 [82–89]	85 [83–89]	0.875
* T4*	84 [80–87]	86 [79–89]	0.413
* T5*	82 [79–87]	84 [80–88]	0.214
** →Average of T1 to T5**	83 [81–88]	84 [81–87]	0.570

Values are presented as median [interquartile range]; SVO2: mixed venous oxygen saturation) T1 = PAC Calibration - T2 = Caval Clamping - T3 = Post Portal Reperfusion - T4 = Post Arterial reperfusion - T5 = End of surgery - pCO2 gradient: Venous-arterial pCO2 gradient (mmHg); SVRI: systemic vascular resistance index

## Discussion

During liver transplantation, the RER had excellent discriminative properties to predict postoperative complications. The RER also had higher sensitivity and specificity than other markers of tissue perfusion, such as the SvO_2_. The only surrogate of tissue perfusion, other than RER, that predicted postoperative complications was blood lactate measured at the end of surgery. This parameter had considerably lower sensitivity and specificity for predicting postoperative complications than did the RER. Our results confirm previous studies in which the potential of the RER to predict postoperative complications has been demonstrated [1, 2], while highlighting the delayed appearance and lower discriminative performance of arterial lactate during liver transplantation.

Lactate has consistently been shown to be an important component for risk stratification in critically ill patients. As it is a product of anaerobic metabolism, many clinicians equate hyperlactatemia with hypoxia. However, hyperlactatemia can arise from non-hypoxemic causes, such as liver disease, and is extremely frequent in liver transplant patients. Nonetheless, hyperlactatemia has been shown to predict primary graft dysfunction and mortality following liver transplantation [12, 13]. Although it remains an essential marker of a mix of tissue hypoxia and liver dysfunction, our results suggest that RER may be more useful to assess intraoperative tissue oxygen delivery and consumption imbalance in this population.

In addition to alterations in lactate metabolism, liver transplant patients suffer from several other physiopathological alterations that may help to explain the lack of sensitivity and specificity of parameters such as VAPCO_2_gap and SvO_2_. VAPCO_2_gap increases exponentially with decreasing cardiac output and has been shown to be extremely useful during hypovolemic, obstructive, and cardiogenic shock [14]. Patients suffering from liver failure, however, exhibit a hyperdynamic state with sustained high cardiac output [15]. Low cardiac output states are quite infrequent in patients suffering from end-stage liver disease and this may explain why the VAPCO_2_gap does not predict postoperative complications in this specific population. SvO_2_ may also be a poor predictor of VO_2_/DO_2_ mismatch in liver transplantation patients due to several reasons, including increased arterio-venous shunting [16] and the hypometabolic effects of anesthesia [17], which both increase SvO_2_.

Previous reports have also shown the capacity of the RER measured specifically during abdominal surgery to predict postoperative complications [12, 13]. This physiological variable could therefore be used in synergy with hemodynamic variables to guide hemodynamic optimization in these patients in the hope of improving tissue oxygenation and lowering the risk of complications. During liver transplantation, measurement of the RER may help confirm the development of tissue hypoxia and the need to correct it by increasing tissue oxygen supply. It may also help differentiate the etiology of hyperlactatemia, a known risk factor for graft dysfunction and mortality. Further studies are required to determine the potential usefulness of this parameter to improve patient outcomes during liver surgery and transplantation using different hemodynamic treatment strategies.

## Limitations

The current study has strengths. Although a retrospective study, intraoperative protocols were standardized and enabled quantification of the RER during key liver transplantation time points. Furthermore, this is the first study of the RER to include only patients undergoing liver transplantation. It gives a clear perspective on this population that poses key perioperative challenges.

This study also had limitations. One important factor we did not measure, since this study was non-interventional, was the time from any corrective treatment or event to an increase in the RER. For this tool to be integrated into a goal-directed hemodynamic strategy, it would be indispensable to know how quickly after an event or treatment the RER worsens or improves. Some potentially interesting variables, such as BIS, central venous pressure, pulmonary artery pressure, or mean infused amount or norepinephrine, were not recorded and could therefore not be analyzed given the retrospective nature of the study. In addition, we did not use the Clavien-Dindo Classification and the Comprehensive Complication Index, which could have been a useful means of comparison for complications.

## Conclusion

During liver transplantation, the RER predicts postoperative complications. This marker may be of particular interest to assess response to hemodynamic alterations and optimization as it is independent of liver function. Implementing this measure intraoperatively may provide a warning for physicians of impending complications and justify more aggressive optimization of oxygen delivery. A large multicenter randomized study is ongoing and will hopefully clarify the situation regarding the interest of using this variable to guide hemodynamic therapy [18].

## Supplementary Information

**Additional file 1 Supplementary Table 1. Intraoperative fluids.**

## Data Availability

The database is closed and there is no public access. However, permission to access and use the database can be obtained if necessary by request to the corresponding author.

## Abbreviations

RER: respiratory exchange ratio
DO_2_: oxygen delivery
VO_2_: oxygen consumption
SvO_2_: Mixed venous oxygen saturation
CO_2_: carbon dioxide
AUC: Area under curve
ROC: receiver operating characteristics
VAPCO_2_gap: veno-arterial difference in the partial pressure of carbon dioxide

## References

[1] Bar S, Grenez C, Nguyen M, de Broca B, Bernard E, Abou-Arab O, Bouhemad B, Lorne E, Guinot PG (2020). Predicting postoperative complications with the respiratory exchange ratio after high-risk noncardiac surgery: a prospective cohort study. Eur J Anaesthesiol.

[2] Bar S, Santarelli D, de Broca B, Abou Arab O, Leviel F, Miclo M, Dupont H, Guinot PG, Lorne E (2021). Predictive value of the respiratory exchange ratio for the occurrence of postoperative complications in laparoscopic surgery: a prospective and observational study. J Clin Monit Comput.

[3] Schumacker PT, Cain SM (1987). The concept of a critical oxygen delivery. Intensive Care Med.

[4] Krafft P, Steltzer H, Hiesmayr M, Klimscha W, Hammerle AF (1993). Mixed venous oxygen saturation in critically ill septic shock patients. The role of defined events. Chest.

[5] Futier E, Robin E, Jabaudon M, Guerin R, Petit A, Bazin JE, Constantin JM, Vallet B (2010). Central venous O2 saturation and venous-to-arterial CO2 difference as complementary tools for goal-directed therapy during high-risk surgery. Crit Care.

[6] Bar S, Nguyen M, de Broca B, Bernard E, Dupont H, Guinot PG (2021). Risk factors and determinants of intraoperative hyperlactatemia in major non-cardiac surgery. J Clin Anesth.

[7] Husain FA, Martin MJ, Mullenix PS, Steele SR, Elliott DC (2003). Serum lactate and base deficit as predictors of mortality and morbidity. Am J Surg.

[8] Vallet B, Blanloeil Y, Cholley B, Orliaguet G, Pierre S, Tavernier B (2013). Guidelines for perioperative haemodynamic optimization. Ann Fr Anesth Reanim.

[9] Karam L, Desebbe O, Coeckelenbergh S, Alexander B, Colombo N, Laukaityte E, Pham H, Lanteri Minet M, Toubal L, Moussa M (2022). Assessing the discriminative ability of the respiratory exchange ratio to detect hyperlactatemia during intermediate-to-high risk abdominal surgery. BMC Anesthesiol.

[10] DeLong ER, DeLong DM, Clarke-Pearson DL (1988). Comparing the areas under two or more correlated receiver operating characteristic curves: a nonparametric approach. Biometrics.

[11] Cannesson M, Le Manach Y, Hofer CK, Goarin JP, Lehot JJ, Vallet B, Tavernier B (2011). Assessing the diagnostic accuracy of pulse pressure variations for the prediction of fluid responsiveness: a "gray zone" approach. Anesthesiology.

[12] Vibert E, Boleslawski E, Cosse C, Adam R, Castaing D, Cherqui D, Naili S, Régimbeau JM, Cunha AS, Truant S (2015). Arterial lactate concentration at the end of an elective hepatectomy is an early predictor of the postoperative course and a potential surrogate of intraoperative events. Ann Surg.

[13] Golse N, Guglielmo N, El Metni A, Frosio F, Cosse C, Naili S, Ichaï P, Ciacio O, Pittau G, Allard MA (2019). Arterial lactate concentration at the end of liver transplantation is an early predictor of primary graft dysfunction. Ann Surg.

[14] Ltaief Z, Schneider AG, Liaudet L (2021). Pathophysiology and clinical implications of the veno-arterial PCO(2) gap. Crit Care.

[15] Fede G, Privitera G, Tomaselli T, Spadaro L, Purrello F (2015). Cardiovascular dysfunction in patients with liver cirrhosis. Ann Gastroenterol.

[16] Parker BM, Dishart MK, Pinsky MR, Kang Y (1999). Is there occult tissue ischemia in chronic end-stage liver disease?. Liver Transpl Surg.

[17] Colonna-Romano P, Horrow JC (1994). Dissociation of mixed venous oxygen saturation and cardiac index during opioid induction. J Clin Anesth.

[18] Bar S, Boivin P, El Amine Y, Descamps R, Moussa M, Abou Arab O, Fischer MO, Dupont H, Lorne E, Guinot PG (2020). Individualized hemodynamic optimization guided by indirect measurement of the respiratory exchange ratio in major surgery: study protocol for a randomized controlled trial (the OPHIQUE study). Trials.

