# Supplementary material for: Intraoperative measurement of the respiratory exchange ratio predicts postoperative complications after liver transplantation
Source: BMC Anesthesiol. 2022 Dec 28;22:405. doi: 10.1186/s12871-022-01949-2 (PMC9795787; doi:10.1186/s12871-022-01949-2)
Supplement: Supplementary file 1 — Additional file 1 Supplementary Table 1. Intraoperative fluids. [file 12871_2022_1949_MOESM1_ESM.docx]

**Supplementary Table 1. Intraoperative fluids**

| **Variables** | **No Complication (n=58)** | **Complication (n=57)** | ***P* value** |
| --- | --- | --- | --- |
| Anesthesia duration (min) | 510 [480-600] | 540 [480-600] | 0.202 |
| Surgical duration (min) | 413 [360-480] | 420 [390-482] | 0.073 |
| Tidal volume (ml) | 480 [450-500] | 500 [460-520] | 0.100 |
| Total crystalloid (ml) | 3500 [2500-5000] | 3500 [2500-5500] | 0.951 |
| Total colloid (ml) | 1500 [1000-2000] | 1500 [1000-2000] | 0.846 |
| Total red blood cells (ml) | 375 [0-1250] | 500 [0-1250] | 0.830 |
| Total platelets (ml) | 0 [0-0] | 0 [0-0] | 0.752 |
| Total fresh frozen plasma (ml) | 500 [0-1000] | 500 [0-1000] | 0.806 |
| Blood loss (ml) | 2000 [1300-3000] | 1700 [1000-3150] | 0.607 |
| Diuresis (ml) | 500 [340-775] | 420 [258-655] | 0.141 |
| Net fluid balance (ml) | 3755 [2650-5200] | 3930 [2867-5180] | 0.524 |
